# A Two Loop Shoelace Granny Knot In The Coronary Sinus

**Published:** 2009-09-01

**Authors:** Gabriel Vanerio

**Affiliations:** CASMU Arrhythmia Service and British Hospital Montevideo, 2420 Av. Italia, Montevideo 1600, Uruguay.

**Keywords:** knot of lead, coronary sinus, CRT

A 62 old-year lady with previous long-standing history of non ischemic dilated cardiomyopathy with an LVEF between 20 to 27%, and complete LBBB underwent a cardiac resynchronization therapy-pacemaker (CRT-P) implantation in another institution. The patient behaved as a non-responder since implant despite AV interval optimization therapy. Chest X-ray (AP and lateral views, Figure 1a and b) was performed that showed a bizarre 'two loop shoelace knot' of the left ventricular lead. The distal portion of the electrode was correctly positioned. The left ventricular R wave sensing was 12 mV, and the left ventricular pacing threshold was 1.5 mV at 0.5 ms, impedance was 643 ohms (at implant was 745 ohms). QRS narrowing was achieved with biventricular ventricular stimulation. A chest CT Scan was performed with no evidence of abnormalities. Several echocardiograms were performed and the CT chest scan showed no evidence of lead malposition within the heart or the mediastinum. No pericardial effusion was observed. We presume that during device implantation when the wire was retrieved, the lead could have adopted these unusual loops. The interesting aspect of the case is the unusual position which the lead took in the coronary sinus while still remaining functional.

## Figures and Tables

**Figure 1a:**
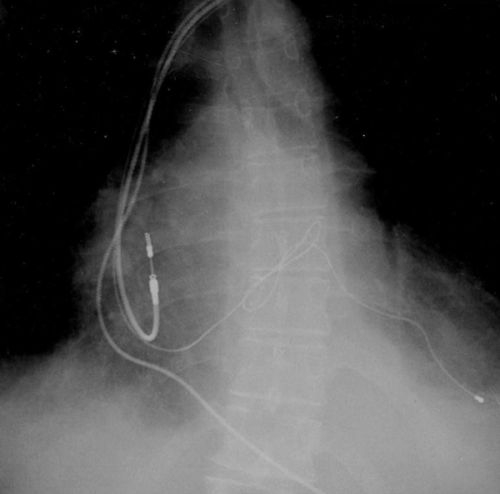
X-ray chest AP view showing 'granny knot' of left ventricular lead in coronary sinus

**Figure 1b:**
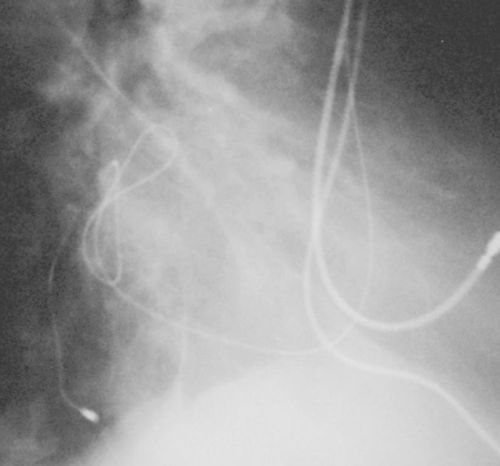
X-ray chest lateral view showing 'granny knot' of left ventricular lead in coronary sinus

